# Effects of Dietary *Salvia sclarea* L. Extract Supplementation on the Gut Microbiota, and Serum Metabolome in Lambs

**DOI:** 10.3390/microorganisms14051163

**Published:** 2026-05-21

**Authors:** Xiaoling Ma, Shanshan Nan, Li Zhang, Yuyang Xue, Wenju Zhang

**Affiliations:** 1 College of Animal Science and Technology, Shihezi University, Shihezi 832000, China; 2 Analysis and Testing Center, Shihezi University, Shihezi 832000, China

**Keywords:** *Salvia sclarea* L. extract, lambs, gut microbiota, metabolome

## Abstract

*Salvia sclarea* L. extract contains various bioactive components such as flavonoids and fatty acids, exhibiting anti-inflammatory, antioxidant, and antibacterial properties. This study aimed to investigate the effects of *Salvia sclarea* L. extract on the gut microbiota and serum metabolome in lambs. Sixty 2-month-old Chinese Merino female lambs (body weight 20 ± 2 kg) were randomly assigned to five groups. The control (CK) group received the basal diet only, while the treatment groups received the basal diet supplemented with 0.04 mL/kg (CL1), 0.08 mL/kg (CL2), 0.12 mL/kg (CL3), and 0.16 mL/kg (CL4) of *Salvia sclarea* L. extract, respectively. The results showed that Firmicutes, Bacteroidetes, Spirochaetes, and Proteobacteria were identified as the dominant phyla across all groups (>90%). Compared with the CK group, CL1 and CL2 groups significantly reduced the relative abundance of Tenericutes (decreased by 38.2% and 32.9%, respectively, *p* < 0.05); the relative abundance of Patescibacteria in the CL1 group was significantly lower (decreased by 55.2%, *p* < 0.05). At the genus level, *Ruminococcaceae* constituted a substantial proportion, including *Ruminococcaceae UCG-005*, *UCG-010*, *UCG-014*, and *NK4A214 group*. STAMP analysis revealed that *Klebsiella* was significantly enriched in CL2, CL3, and CL4 groups compared to the CK group (*p* < 0.05). Correlation analysis between microbiota and immune indices showed that *Christensenellaceae R-7 group* was significantly negatively correlated with TNF-α (*p* < 0.05); *Ruminococcaceae UCG-005* was significantly negatively correlated with IFN-γ (*p* < 0.05) and showed a negative correlation trend with immunoglobulins (IgA, IgG, IgM). Conversely, *Ruminococcaceae UCG-014* was significantly positively correlated with IL-4 (*p* < 0.05) but showed a negative correlation trend with IgM. Untargeted metabolomics analysis identified 8, 18, 25, and 20 differential metabolites in CL1, CL2, CL3, and CL4 groups, respectively. Notably, 3-hydroxy-7-methoxyflavone and Gamma-Glu-Cys were significantly upregulated across all treatment groups. KEGG pathway enrichment analysis indicated that these differential metabolites were primarily involved in nucleotide metabolism, fatty acid biosynthesis, and oxidative stress-related pathways. Further Spearman correlation analysis revealed significant associations between gut microbiota and differential metabolites. Specifically, *g_Klebsiella* was significantly positively correlated with 3-Hydroxycapric acid and 3-hydroxy-7-methoxyflavone (*p* < 0.05). In conclusion, *Salvia sclarea* L. extract modulates host energy metabolism by regulating nucleotide metabolism and fatty acid biosynthesis, and enhances immune function by alleviating oxidative stress, through the remodeling of gut microbiota and serum metabolome.

## 1. Introduction

The quality of early growth and development in lambs directly determines adult productivity and health status. Early weaning is a critical management practice in modern sheep production, aimed at improving ewe reproductive efficiency and reducing rearing costs [1]. However, neonatal ruminants lack a fully functional rumen at birth, and their digestive physiology highly depends on the synergistic interaction between gut development and microbial colonization [2]. Weaning stress is frequently accompanied by disruptions in gut microbial communities, impaired barrier function, and immunosuppression, leading to reduced growth performance and increased disease susceptibility in lambs. As a “microbial organ” of the host, the gut microbiota plays a central role in maintaining host metabolic homeostasis by fermenting dietary fiber to produce short-chain fatty acids (SCFAs), synthesizing vitamins, and regulating immune system maturation [3]. Therefore, exploring safe and effective gut microecological regulation strategies is of great significance for alleviating weaning stress and promoting healthy lamb production.

Plant extracts have emerged as promising green feed additives due to their natural origin, multi-target effects, and low residue characteristics. *Salvia sclarea* L., an aromatic plant belonging to the genus *Salvia* of the *Lamiaceae* family, is rich in volatile oils (such as linalool and linalyl acetate), flavonoids, and terpenoids in its extracts, exhibiting anti-inflammatory, antioxidant, antimicrobial, and neuromodulatory bioactivities [4]. Previous studies have shown that *Salvia sclarea* L. extract can improve host metabolic health, but the specific regulatory mechanisms on the gut microecology of ruminants remain unclear. Based on preliminary findings, *Salvia sclarea* L. extract significantly affects the growth performance and immune indicators of lambs [5]; this study hypothesized that this extract may modulate host metabolism through optimization of gut microbial profiles. Therefore, this experiment combined 16S rRNA sequencing with untargeted metabolomics to systematically investigate the effects of different doses of *Salvia sclarea* L. extract on the fecal microbiota structure and serum metabolome of lambs, and further analyzed the correlations between microbes and metabolites, aiming to provide a theoretical basis for the development of novel green functional feed additives.

## 2. Materials and Methods

All animal experimental protocols were conducted in strict compliance with the relevant guidelines and were authorized by the Institutional Animal Bioethics Committee of Shihezi University (Xinjiang, China).

### 2.1. Experiment Material

The *Salvia sclarea* L. extract (essential oil) used in the experiment appeared to be a light-yellow liquid. It was prepared by hydrodistillation of the flowers, leaves, and stems of *Salvia sclarea* L. The active ingredients were linalyl acetate (54.79%) and linalool (30.22%), as detected using GC–MS. The purity of the *Salvia sclarea* L. extract was 85%. It was produced in the East Industrial Park of Zhaosu County, Xinjiang, China (Xinjiang Tianshan Salvia Sclarea Aromatics Co., Ltd.).

### 2.2. Experimental Design, Animals, and Management

Sixty female Chinese Merino lambs (2 months old; 20 ± 2 kg BW) were randomly divided into 5 groups (*n* = 12 per group). For the subsequent microbiome and metabolomics analyses, 8 biological replicates per group were included. The remaining 4 lambs per group were excluded prior to sequencing and metabolomic assays based on strict exclusion criteria, which included abnormal physiological performance, insufficient sample quality, and statistical outlier screening. The Control group (CK) was fed a basal diet only. The *Salvia sclarea* L. extract groups received the basal diet supplemented with graded levels of the extract: 0.04 mL (CL1), 0.08 mL (CL2), 0.12 mL (CL3), and 0.16 mL (CL4) per kilogram of basal diet (mL/kg diet), respectively. The feeding trial lasted for 85 days, comprising a 15-day adaptation period followed by a 70-day experimental period. The experimental sheep were fed twice daily at 06:00 and 18:00. The *Salvia sclarea* L. extract was precisely and uniformly mixed with the basal diet according to the experimental design. The *Salvia sclarea* L. extract was first pre-mixed with a small amount of concentrate, and then thoroughly blended into the total diet by stepwise scale-up mixing. The sheep were housed in individual cage enclosures (1.5 × 1 × 1 m) with free access to water. The animal sheds, water troughs, and feed bunks were cleaned and disinfected routinely throughout the experiment. The ingredient composition and nutrient levels of the basal diet are presented in Table 1.

### 2.3. Sample Collection

On the final day of the experiment, before the morning feeding, fasting blood samples were collected from the jugular vein of each lamb into 10 mL Vacutainer tubes containing EDTA. The samples were immediately centrifuged at 3000× *g* for 15 min at 4 °C to separate serum, which was then stored at −80 °C pending UPLC-Q-TOF/MS analysis. Fecal samples were collected transrectally using disposable PE gloves, which were changed for each lamb to prevent cross-contamination. Approximately 2 g of fresh feces were collected using a sterile scoop and transferred into 2 mL sterile cryovials. The samples were immediately snap-frozen in liquid nitrogen and subsequently stored at −80 °C for subsequent sequencing analysis.

### 2.4. 16S rRNA Gene Sequencing and Analysis

Microbial diversity in fecal samples was assessed by 16S rRNA gene high-throughput sequencing. Total genomic DNA was extracted using the QIAamp PowerFecal Pro DNA Kit (QIAGEN, Hilden, Germany). DNA integrity and concentration were evaluated by 1% agarose gel electrophoresis and NanoDrop 2000 spectrophotometry (Thermo Fisher Scientific, Waltham, MA, USA), respectively. Following quality verification, the V3–V4 hypervariable region of the 16S rRNA gene was amplified using universal primers 338F and 806R. The resulting amplicons were used to construct sequencing libraries, which were then subjected to paired-end sequencing on the Illumina MiSeq PE250 platform (Illumina, San Diego, CA, USA). Raw sequencing reads were subjected to rigorous quality control, including adapter trimming, chimera removal, and elimination of low-quality reads, to generate high-quality clean data. Subsequently, operational taxonomic units (OTUs) were clustered at 97% sequence similarity using QIIME (v1.8.0), and representative sequences were taxonomically annotated against the SILVA reference database to characterize the microbial community composition.

### 2.5. Untargeted Metabolomics Analysis

Serum samples were thawed at 4 °C, and 100 μL aliquots were mixed with 400 μL pre-cooled methanol/acetonitrile (1:1, *v*/*v*) for protein precipitation. The mixture was vortexed and centrifuged at 14,000× *g* for 20 min at 4 °C. The resulting supernatant was collected and vacuum-dried. For LC-MS analysis, the dried residues were reconstituted in 100 μL acetonitrile/water (1:1, *v*/*v*). After vortexing, the solution was centrifuged again at 14,000× *g* for 20 min at 4 °C. The final supernatant was transferred to LC vials for UHPLC-MS/MS analysis. LC-MS/MS analysis was performed on a 1290 Infinity UHPLC system (Agilent Technologies, Santa Clara, CA, USA) coupled with a TripleTOF 6600 mass spectrometer (AB Sciex, Framingham, MA, USA). Chromatographic separation was achieved on an ACQUITY UPLC BEH C18 column (2.1 mm × 100 mm, 1.7 μm; Waters, Milford, CT, USA). Mass spectrometric data were acquired in data-dependent acquisition (DDA) mode, consisting of a full-scan survey (*m*/*z* 70–1050) followed by MS/MS fragmentation. The instrument was operated in both positive and negative electrospray ionization (ESI) modes with the following source parameters: ion source gas 1 (Gas 1) and ion source gas 2 (Gas 2) at 60 psi, curtain gas (CUR) at 30 psi, source temperature at 600 °C, and ion spray voltage floating (ISVF) at ±5500 V.

LC/MS raw data were preprocessed using Progenesis QI (version 3.0, Waters Corporation, Milford, MA, USA) software. Internal standard peaks, as well as any known false positive peaks (including noise, column bleed, and derivatized reagent peaks), were removed from the data matrix, and the data were then de-redunded and peak-pooled. At the same time, the metabolites were identified by searching the databases. The main databases were HMDB (http://www.hmdb.ca/) Database. Metabolite features detected in ≥80% of samples were retained. Data were normalized by sum normalization, log10-transformed, and batch-corrected after removal of QC samples with RSD > 30%. OPLS-DA (R package “ropls”, version 1.6.2) was used to identify differential metabolites (VIP > 1, *p* < 0.05, unadjusted) using 7-fold cross-validation. After quality control and preprocessing of the metabolomic raw data, differential metabolites were annotated in KEGG, screened, identified, and subjected to KEGG pathway enrichment analysis. Metabolite profiling was initially conducted in both ESI positive and negative ionization modes. Considering the higher number of annotated metabolites, better separation performance and more stable repeatability, only the ESI negative ion mode data were used for subsequent differential metabolite analysis and result presentation, while the positive ion mode data were not displayed in this study.

### 2.6. Statistical Analysis

To investigate the fecal microbial community structure and serum metabolic profiles across different experimental groups, taxonomic abundance differences were analyzed using STAMP (version 2.1.3), while microbial community dissimilarities were assessed by Adonis analysis based on Bray–Curtis distance matrices. Subsequently, the top ten most abundant microbes were correlated with growth, metabolic, and immune indices using Spearman correlation analysis. For metabolomics, significantly differential metabolites were identified using the criteria of VIP > 1 and *p* < 0.05. Furthermore, Spearman correlation analysis was applied to integrate 16S rRNA sequencing data with metabolomics data, aiming to explore the associations between significantly differential microbial taxa and serum metabolites. All data visualization and graphical plotting were conducted using the bioinformatics cloud platform provided by APT Bio (Zhongke New Life, Shanghai, China).

## 3. Results

### 3.1. Sequencing Quality Control

After quality filtering and chimera removal, all samples obtained high-quality sequencing data. The effective raw reads of each sample ranged from 80,113 to 12,1751, with total bases ranging from 33.01 Mb to 49.97 Mb. The Q20 rate of all samples was between 98.30 and 98.49%, and the Q30 rate ranged from 94.51 to 95.00%. The GC content was distributed steadily among samples, indicating that the sequencing quality was high, stable, and met the requirements for subsequent microbiome bioinformatics analysis (Supplementary Table S1).

### 3.2. Analysis of Alpha Diversity of Fecal Microbiota in Lambs

**Figure 1:**
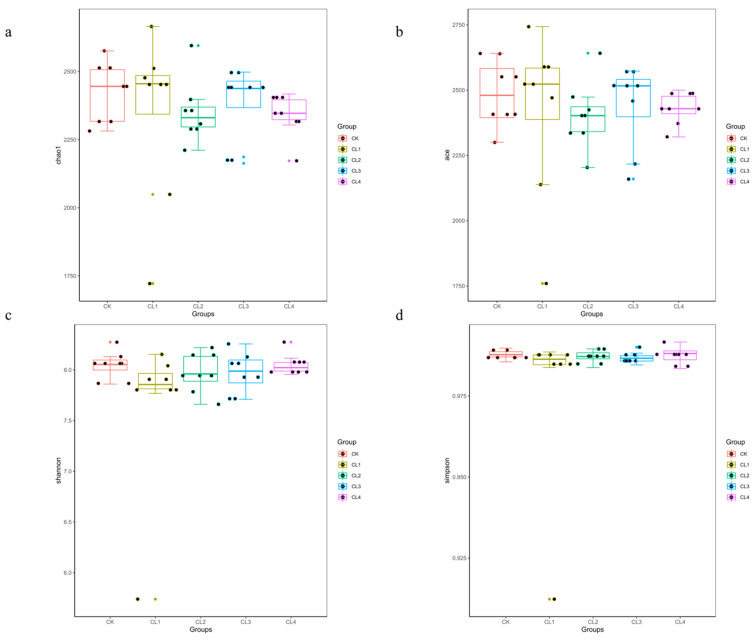
Alpha diversity of fecalmicrobiota in lambs under different *Salvia sclarea* L. extract concentrations. (**a**) ACE index, (**b**) Chao1 index, (**c**) Shannon index, (**d**) Simpson index. Data are based on eight biological replicates per group (*n* = 8).

To analyze the effects of different concentrations of *Salvia sclarea* L. extract on the alpha diversity of the fecal microbiota in lambs, we calculated the Ace, Chao1, Shannon, and Simpson indices. The results showed no significant differences in richness indices (ACE, Chao1) or diversity indices (Shannon, Simpson) between the treatment groups and the control group (*p* > 0.05, Figure 1).

### 3.3. Relative Abundance of Fecal Microbiota at the Phylum Level in Lambs

**Figure 2:**
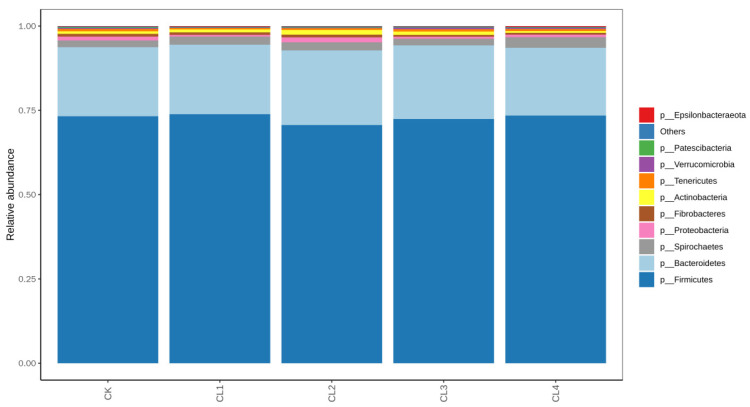
Effect of *Salvia sclarea* L. extract on the relative abundance of fecal microbiota at the phylum level in lambs (*n* = 8 per group).

The impact of *Salvia sclarea* L. extract on the relative abundance of fecal bacterial phyla in lambs is presented in Figure 2. The dominant Phylums across all groups were Firmicutes, Bacteroidetes, Spirochaetes, and Proteobacteria, collectively accounting for over 90% of the total relative abundance. Supplementation with *Salvia sclarea* L. extract significantly altered the abundance of specific Phylum (*p* < 0.05). Compared with the control group, CL1 and CL2 groups significantly reduced the relative abundance of Tenericutes (decreased by 38.2% and 32.9%, respectively, *p* < 0.05), the relative abundance of Patescibacteria in the CL1 group was significantly lower (decreased by 55.2%, *p* < 0.05). Additionally, Verrucomicrobia was significantly more abundant in CL3 and CL4 groups than in CL1 and CL2 groups.

### 3.4. Relative Abundance of Fecal Microbiota at the Genus Level in Lambs

**Figure 3:**
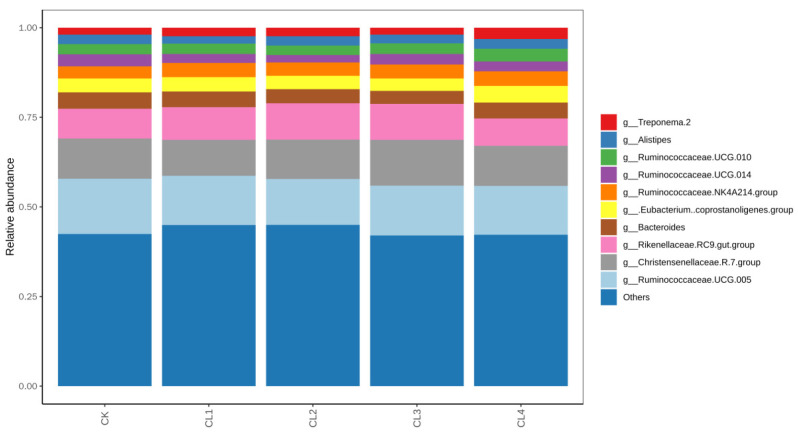
Effect of *Salvia sclarea* L. extract on the relative abundance of fecal microbiota at the genus level in lambs (*n* = 8 per group).

The predominant genera across all groups included *Ruminococcaceae UCG-005*, *Christensenellaceae R-7 group*, and *Rikenellaceae RC9 gut group*, which collectively accounted for a high proportion of the total relative abundance (Figure 3). No significant differences were observed in the relative abundances of these dominant genera among the treatment groups (*p* > 0.05). However, the supplementation of *Salvia sclarea* L. extract significantly influenced the abundances of certain non-dominant genera. Specifically, the abundance of *Ruminococcaceae UCG-010* was significantly higher in the CL4 group compared to the CL2 group (*p* < 0.05), while the abundance of *Ruminococcaceae UCG-014* was significantly lower in the CL2 group than in the CK group (*p* < 0.05). These findings indicate that although *Salvia sclarea* L. extract had no significant effect on the overall fecal microbiota community structure at the genus level, it exerts a selective modulatory effect on the abundance of specific non-dominant genera.

### 3.5. Differential Fecal Microbiota at the Genus Level in Lambs

**Figure 4:**
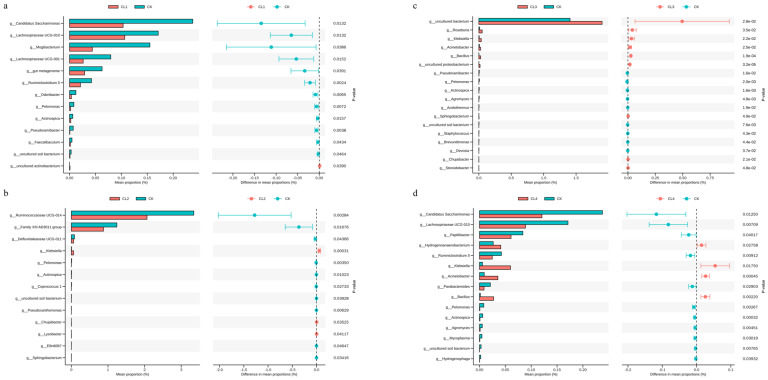
STAMP analysis of fecal microbial composition at the genus level in lambs supplemented with different concentrations of *Salvia sclarea* L. extract. The left panels display the mean proportion (%) of specific taxa, while the right panels show the difference in mean proportions with 95% confidence intervals (indicated by error bars). Data are based on eight biological replicates per group (*n* = 8). (**a**) CK vs. CL1, (**b**) CK vs. CL2, (**c**) CK vs. CL3, (**d**) CK vs. CL4.

STAMP analysis revealed significant differences in the fecal microbial community structure at the genus level between the different *Salvia sclarea* L. extract dosage groups and the CK group, exhibiting a dose-dependent regulatory pattern (Figure 4). Compared with the CK group, the CL1 group showed significantly decreased relative abundances of 12 genera, including *Candidatus Saccharimonas*, *Lachnospiraceae UCG-010*, and *Mogibacterium*, while only *uncultured* *actinobacterium* was significantly enriched (*p* < 0.05). In the CL2 group, the relative abundances of *Ruminococcaceae UCG-014*, *Defluviitaleaceae UCG-011*, and *Sphingobacterium* were significantly reduced, whereas eight genera, including *Klebsiella*, *Pelomonas*, and *Actinospica*, were significantly increased (*p* < 0.05). The CL3 group displayed significantly elevated levels of *uncultured bacterium*, along with 13 enriched genera including *Roseburia* and *Klebsiella* (*p* < 0.05). Similarly, the CL4 group exhibited significantly lower abundances of seven genera, including *Candidatus Saccharimonas* and *Lachnospiraceae UCG-010*, but significantly higher abundances of nine genera, including *Hydrogenoanaerobacterium* and *Klebsiella* (*p* < 0.05). Notably, *Klebsiella* was significantly enriched across the CL2, CL3, and CL4 groups, suggesting it may be a core responsive genus to *Salvia sclarea* L. extract supplementation. In conclusion, *Salvia sclarea* L. extract exerts a dose-dependent, bidirectional regulatory effect on the fecal microbiota of lambs. While low doses primarily inhibit specific genera, moderate-to-high doses present a complex pattern of concurrent inhibition and promotion. *Salvia sclarea* L. extract selectively modulates the fecal microbial community primarily through suppressing specific potentially harmful or competitive genera while concomitantly promoting the proliferation of particular uncultured taxa.

### 3.6. Gut Microbiota at the Genus Level Correlates with Serum Immune Indices in Lambs

**Figure 5:**
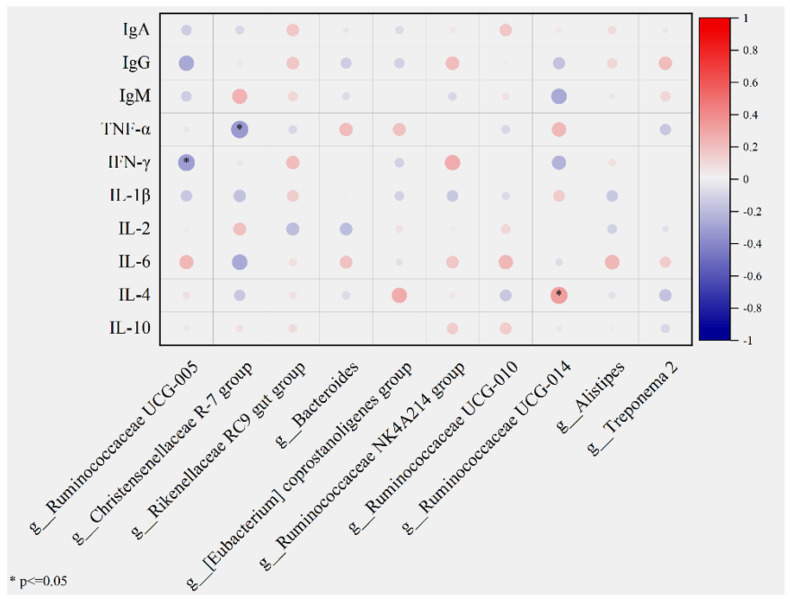
Correlation analysis between fecal microorganisms and serum antioxidant and immune-related indicators. The color scale ranges from blue (−1) to red (1), representing negative and positive correlations, respectively. The size of the circle corresponds to the strength of the correlation. Asterisks (*) indicate statistically significant correlations (*p* ≤ 0.05).

Correlation analysis between fecal microbiota at the genus level and serum immune indices in lambs is presented in Figure 5. The results revealed significant associations between specific bacterial genera and immune factors (*p* < 0.05). Specifically, *Christensenellaceae R-7 group* exhibited a significant negative correlation with Tumor Necrosis Factor-α (TNF-α) (*p* < 0.05), along with negative trends for interleukin (IL-1β, IL-4 and IL-6). *Ruminococcaceae UCG-005* showed a significant negative correlation with interferon-γ (IFN-γ) (*p* < 0.05), as well as negative trends with immunoglobulins (IgA, IgG, IgM). Conversely, *Ruminococcaceae UCG-014* was significant positive correlation with IL-4 (*p* < 0.05), but showed a negative trend with IgM. Additionally, positive correlations were observed between *Ruminococcaceae NK4A214 group* and IFN-γ, while *Rikenellaceae RC9 gut group* was negatively correlated with IL-2. These findings suggest that specific fecal bacterial genera may participate in the regulation of host immune homeostasis by modulating the balance of pro- and anti-inflammatory cytokines as well as immunoglobulin secretion.

### 3.7. Chemical Superclass Classification of Identified Metabolites

**Figure 6:**
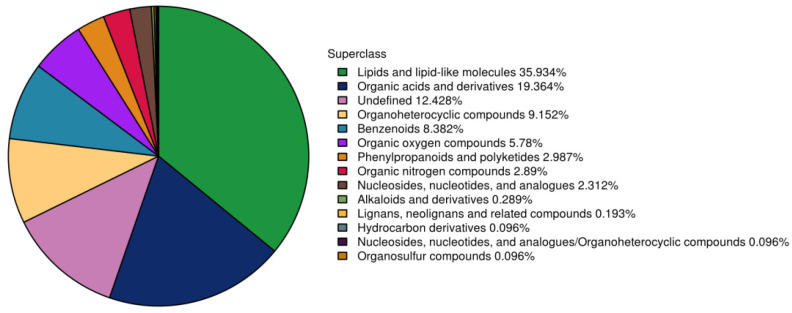
Distribution of identified metabolites across different chemical superclasses. The pie chart illustrates the distribution of metabolites across different chemical superclasses based on the HMDB database.

Chemical superclass classification of the identified metabolites revealed a diverse composition, predominantly enriched in Lipids and Organic acids (Figure 6). Specifically, 373 metabolites were assigned to Lipids and lipid-like molecules, accounting for 35.93% of the total, followed by Organic acids and derivatives (201 metabolites, 19.36%). Collectively, these two classes exceeded 55% of the total abundance, indicating that they are the predominant metabolic components in the samples. Additionally, Organoheterocyclic compounds and Benzenoids accounted for 9.15% and 8.38%, respectively, while Organic oxygen compounds accounted for a certain proportion (5.78%). In summary, the metabolite profile was dominated by Lipids and lipid-like molecules, with Organic acids and derivatives as the secondary major class, reflecting the metabolic characteristics of the samples.

### 3.8. Multivariate Statistical Analysis of Metabolic Profiles

**Figure 7:**
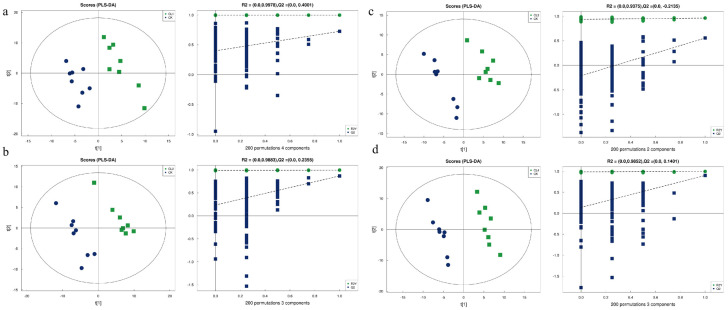
PLS-DA analysis and permutation tests of differential serum metabolites in negative ion mode. The left panels show the PLS-DA score plots, and the right panels show the permutation tests for the model validation. (**a**) CK vs. CL1, (**b**) CK vs. CL2, (**c**) CK vs. CL3, and (**d**) CK vs. CL4. The R^2^ and Q^2^ values indicate the goodness of fit and predictability of the model, respectively. Each group consists of eight biological replicates (*n* = 8).

The PLS-DA score plots revealed a distinct separation trend between the treatment groups (CL1, CL2, CL3, and CL4 groups) and the CK group along the principal component axis (Figure 7). The treatment samples were primarily aggregated in the positive region, whereas the CK group was distributed in the negative region, indicating that different addition levels induced significant metabolic alterations. To verify model reliability, permutation tests (*n* = 200) were conducted. All models exhibited R^2^ values close to 1.0, indicating high explanatory capacity. Furthermore, the intercept of the Q^2^ regression line on the y-axis was less than 0.4, and R^2^ was greater than Q^2^, indicating that the models were not overfitted. Consequently, the models are considered robust and reliable for subsequent analysis.

### 3.9. Differential Analysis of Serum Metabolites

**Figure 8:**
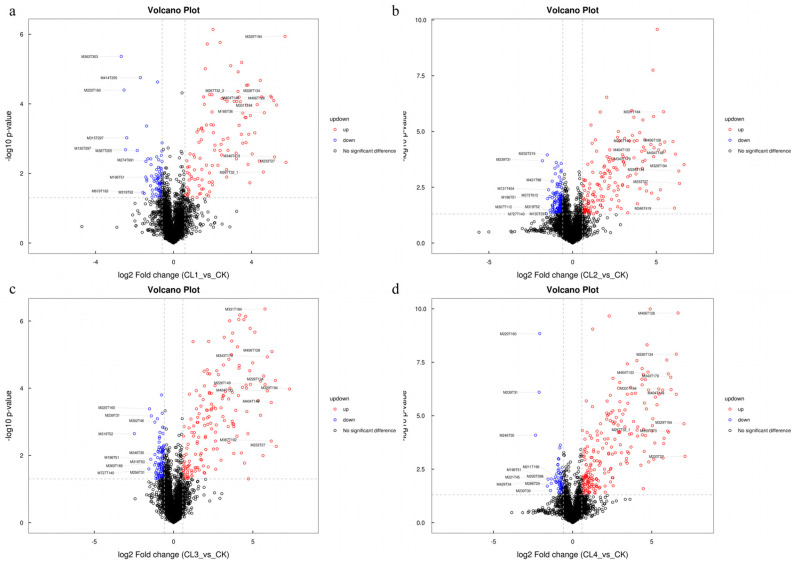
Volcano plot of differential serum metabolites under negative ion mode for CK vs. CL1 (**a**), CK vs. CL2 (**b**), CK vs. CL3 (**c**), and CK vs. CL4 (**d**). Significantly upregulated and downregulated metabolites are highlighted in red and blue, respectively, based on a threshold of *p* < 0.05 and VIP > 1. The vertical dashed lines represent the fold change threshold (|log2 Fold Change| > 1), and the horizontal dashed line represents the statistical significance threshold (−log10 *p*-value > 1.3, corresponding to *p *< 0.05).

Based on the PLS-DA model, differential analysis was performed on metabolites detected in negative ion mode, and the results were visualized using volcano plots (Figure 8). Using a VIP > 1 and *p* < 0.05 as the criteria for significant difference, the results showed that compared with the CK group, 8, 18, 25, and 20 significantly differential metabolites were identified in the CL1, CL2, CL3, and CL4 groups, respectively. In the CL1 group, the differential metabolites were primarily composed of flavonoids and amino acid derivatives. Notably, 3-hydroxy-7-methoxyflavone (FC = 22.80) and Gamma-Glu-Cys (FC = 11.12) were significantly upregulated, suggesting that low concentrations of *Salvia sclarea* L. extract can activate the antioxidant defense system. In addition, 3-Hydroxycapric acid and Inosine also showed significant upward trends. The significant upregulation of these metabolites indicates that specific metabolic pathways—such as flavonoid biosynthesis, glutathione metabolism, or purine metabolism—may be activated to trigger cellular stress responses or functional regulation. Meanwhile, 4 metabolites were significantly downregulated in this group, including Lithosprmoside, L-methionine, and Phenaceturic acid. The downregulation of these substances may be associated with decreased antioxidant capacity or inhibition of amino acid metabolic pathways, reflecting early responses of primary and secondary metabolism. In the CL2 group, 10 upregulated and 8 downregulated metabolites were identified. The metabolites already upregulated in CL1 (3-hydroxy-7-methoxyflavone, Gamma-Glu-Cys, Inosine, and 3-Hydroxycapric acid) remained elevated in CL2. Furthermore, metabolites such as Zanamivir, Physcion, and Bonactin were also significantly upregulated. Notably, the long-chain fatty acid metabolites Palmitic acid (FC = 0.78) and Oleic acid (FC = 0.74) were significantly downregulated, implying that treatment at this concentration may inhibit lipid metabolism. Concurrently, the significant downregulation of Indoxylsulfate and Thymine may interfere with fecal microbial pyrimidine nucleotide metabolism.

The CL3 group exhibited the highest number of differential metabolites, indicating that treatment at this concentration has the most extensive impact on the organism’s metabolism. The core differential metabolites, 3-hydroxy-7-methoxyflavone and Gamma-Glu-Cys, maintained a significant upregulation, demonstrating that antioxidant and flavonoid metabolic pathways remain highly activated at this concentration. Notably, steroid hormone metabolites 5α-pregnan-3α,17-diol-20-one 3-sulfate (FC = 13.76) and Estrone sulfate (FC = 4.25) were significantly upregulated. Additionally, medium-chain fatty acids (such as 3-Hydroxycapric acid and Capric acid) and amino acid derivatives (N-acetyl-D-galactosaminitol) were also significantly upregulated, reflecting a reprogramming of energy and amino acid metabolism. The downregulated metabolites were predominantly polyunsaturated fatty acid derivatives, including Octadecanoic acid, 12(R)-HETE, Prostaglandin I2, 11-dehydrothromboxane B2, and Ricinoleic acid, which are inflammatory lipid mediators. In the CL4 group, β-D-allose (FC = 67.01) emerged as the hallmark upregulated metabolite, while 3-hydroxy-7-methoxyflavone and Gamma-Glu-Cys remained at extremely high levels. Steroid hormone metabolites, including 5α-pregnan-3α,17-diol-20-one 3-sulfate and Estrone sulfate, were significantly enriched. Furthermore, metabolites such as 3-Hydroxycapric acid, Zanamivir, and N-acetyl-D- galactosaminitol maintained a continuous upward trend in the CL4 group. In contrast, various plant-derived natural products were significantly downregulated, including Cinnamoylglycine, Embelin, Ganoderic acid A, and Dihydromyricetin, suggesting that the synthesis of specific secondary metabolites was impeded or that specific catabolic pathways were inhibited. Notably, 3-hydroxy-7-methoxyflavone and Gamma-Glu-Cys were commonly highly upregulated across all four treatment groups, constituting the core biomarkers of the metabolic influence exerted by *Salvia sclarea* L. extract.

### 3.10. Enrichment Characteristics of Differentially Expressed Metabolites in KEGG Pathways

**Figure 9:**
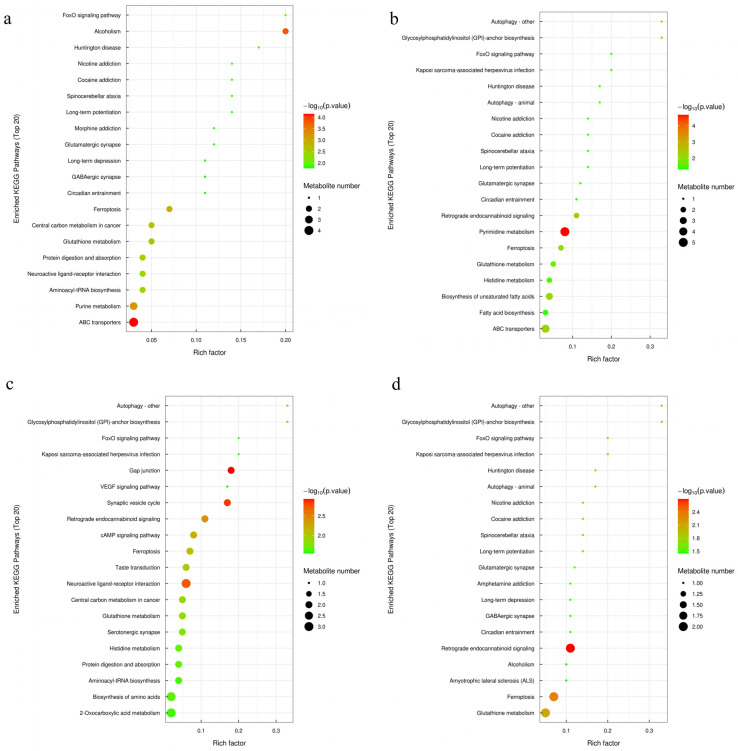
Bubble plot of KEGG pathway enrichment analysis for differential metabolites. (**a**) CK vs. CL1, (**b**) CK vs. CL2, (**c**) CK vs. CL3, (**d**) CK vs. CL4. The color of the dots represents the *p*-value (ranging from green to red, indicating decreasing statistical significance), and the size of the dots represents the number of enriched metabolites in each pathway.

KEGG pathway enrichment analysis revealed that *Salvia sclarea* L. extract treatment induced a dose-dependent metabolic pathway activation pattern (Figure 9). Compared with the CK group, differential metabolites in the CL1 group were primarily enriched in ABC transporters, Purine metabolism, and oxidative stress-related pathways( Ferroptosis and Glutathione metabolism). Additionally, fatty acid biosynthesis, FoxO signaling pathway, and autophagy-related pathways were significantly activated, suggesting that low-concentration treatment mainly promotes substance transport, energy metabolism, and basic cellular defense mechanisms. The KEGG pathway enrichment pattern in the CL2 group was similar to that of CL1, but Pyrimidine metabolism replaced Purine metabolism as the most significantly enriched nucleotide metabolic pathway. Moreover, especially regarding the enrichment factors of Retrograde endocannabinoid signaling, biosynthesis of unsaturated fatty acids, and Glycosylphosphatidylinositol (GPI)-anchor biosynthesis were higher than those in CL1 group. The CL3 group exhibited prominent neuroregulatory features, with Gap junction and Synaptic vesicle cycle showing the highest enrichment levels. Concurrently, Retrograde endocannabinoid signaling, cAMP signaling pathway, and Neuroactive ligand-receptor interaction were significantly activated. Neuro-related pathways dominated in the CL4 group, with Retrograde endocannabinoid signaling showing the highest enrichment degree; Glutamatergic and GABAergic synapses were synchronously activated, accompanied by enrichment of long-term potentiation/depression pathways, whereas the enrichment degrees of oxidative stress pathways Ferroptosis and Glutathione metabolism were lower than those in CL1/CL2 groups. In summary, the regulation of serum metabolism in lambs by *Salvia sclarea* L. extract showed a clear dose-dependent transition: from basic metabolism (nucleotide and fatty acid synthesis) and oxidative stress defense at low concentrations, gradually transitioning to neurosignal regulation at medium-high concentrations, and ultimately manifesting as significant intervention in neurotransmitter systems and synaptic function at high concentrations.

### 3.11. Spearman Correlation Analysis

**Figure 10:**
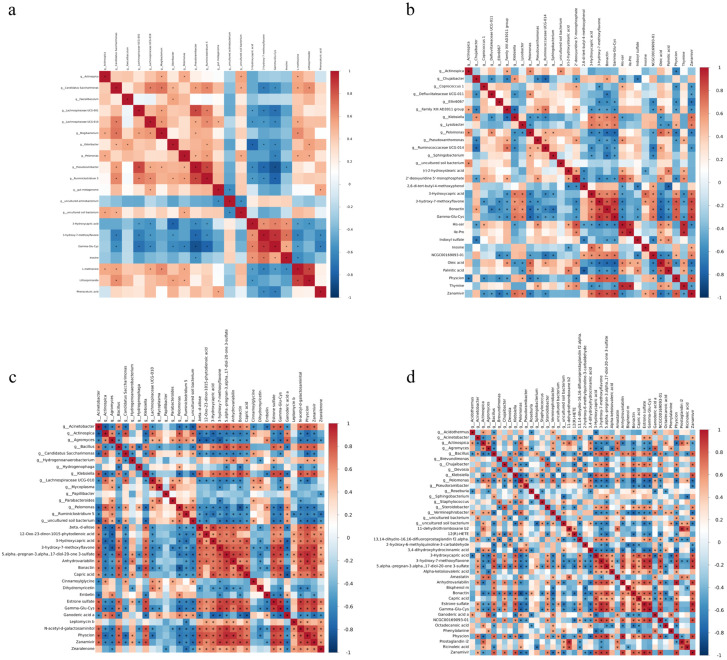
Correlation analysis between fecal microbiota and serum metabolites. (**a**) CK vs. CL1, (**b**) CK vs. CL2, (**c**) CK vs. CL3, (**d**) CK vs. CL4. Correlation strength is indicated by color intensity, with red representing positive correlations and blue representing negative. Asterisks (*) indicate statistically significant correlations (*p* < 0.05).

To investigate the effects of different concentrations of *Salvia sclarea* L. extract on the interaction between fecal microecology and host metabolism in lambs, this study employed Spearman correlation analysis to construct microbe-metabolite correlation networks for each treatment group (CL1–CL4 group) and the CK group (Figure 10). The results revealed extensive association patterns between differential bacterial genera and serum differential metabolites across all groups, with the complexity and specificity of the correlation networks gradually increasing as the concentration of *Salvia sclarea* L. extract increased.

Compared with the CK group, the CL1 group exhibited a significant modular co-occurrence pattern between the microbiota and metabolites. Specific dominant bacterial genera represented by *g_Actinospica*, *g_Candidatus Saccharimonas*, and *g_Faecalibaculum* showed significant positive correlations with serum metabolites such as Phenaceturic acid and Lithospermoside (*p* < 0.05), suggesting that these microbiota may promote the accumulation of related metabolites. Conversely, these genera exhibited strong negative correlations with metabolites including 3-Hydroxycapric acid and 3-hydroxy-7-methoxyflavone (*p* < 0.05), indicating that these microbiota may initiate host antioxidant defense responses under low-concentration *Salvia sclarea* L. extract treatment by participating in flavonoid metabolism and glutathione synthesis pathways. Furthermore, bacterial genera such as *g_Odoribacter*, *g_Pelomonas*, and *g_Pseudoramibacter* showed significant negative correlations with energy metabolism-related metabolites like 3-Hydroxycapric acid and Inosine, while displaying positive correlations with downregulated metabolites such as L-methionine, Phenaceturic acid, and Lithospermoside, reflecting the microbial regulation of basal energy metabolism.

The complexity of the association network between microbiota and metabolites in the CL2 group increased significantly, exhibiting a denser pattern of positive and negative interactions. Specifically, *g_Klebsiella* showed a significant positive correlation with upregulated metabolites such as 3-Hydroxycapric acid, 3-hydroxy-7-methoxyflavone, Bonactin, and Gamma-Glu-Cys, while displaying a significant negative correlation with the downregulated Oleic acid (*p* < 0.05), suggesting the central role of this genus in lipid metabolic remodeling. The *g_Lysobacter* demonstrated significant positive correlations with the aforementioned upregulated metabolites, as well as with NCGC00169093-01 and Zanamivir, and exhibited negative correlations with Oleic acid and Palmitic acid (*p* < 0.05), indicating its synergistic participation in the regulation of antioxidant and energy metabolism. Furthermore, *g_Pelomonas* showed significant negative correlations with 3-Hydroxycapric acid, 3-hydroxy-7-methoxyflavone, Bonactin, and Gamma-Glu-Cys, while displaying positive correlations with Oleic acid and Palmitic acid (*p* < 0.05).

Compared with the CK group, *g_Klebsiella*, *g_Staphylococcus*, and *g_Steroidobacter* in the CL3 group exhibited significant positive correlations with metabolites such as 3-hydroxy-7-methoxyflavone, 5α-pregnan-3α,17-diol-20-one 3-sulfate, Estrone sulfate, and Gamma-Glu-Cys. This suggests that these genera synergistically participate in steroid hormone synthesis, antioxidant defense, and energy metabolism. Conversely, *g_Actinospica*, *g_Agromyces*, *g_Pelomonas*, *g_Pseudoramibacter*, and *g_Verminephrobacter* showed significant negative correlations with the aforementioned metabolites, indicating that *Salvia sclarea* L. extract inhibits these genera, thereby reducing the consumption or interference of key metabolites. Notably, the anti-inflammatory lipid mediators Prostaglandin i2 and Ricinoleic acid were negatively correlated with *g_Roseburia* and *g_Staphylococcus*, suggesting that *Salvia sclarea* L. extract may exert anti-inflammatory effects by suppressing the pro-inflammatory microbiota-metabolite axis. Furthermore, the negative correlation between *g_Roseburia* and Ganoderic acid, as well as Octadecanoic acid, indicates its synergistic involvement in the metabolic transformation of long-chain fatty acids and exogenous bioactive substances.

Spearman correlation analysis revealed the association patterns between fecal microbiota and serum metabolites in the CL4 group (Figure 10). The results showed that genera such as *g_Acinetobacter*, *g_Bacillus*, and *g_Klebsiella* were significantly positively correlated with metabolites including 3-Hydroxycapric acid, and 3-hydroxy-7-methoxyflavone, but negatively correlated with natural products such as Ganoderic acid a, Embelin, and Dihydromyricetin. This suggests a core regulatory role for these genera under high-concentration treatment, where they may synergistically promote the synthesis of fatty acid derivatives and plant-derived metabolites. The positive correlation between *g_Hydrogenophaga* and metabolites such as Anhydroviridibillin and 5-alpha-pregnan-3-alpha,17-diol-20-one 3-sulfate implies its potential involvement in regulating host steroid metabolic pathways. Conversely, genera like *g_Actinospica* and *g_Agromyces* exhibited significant negative correlations with metabolites such as Cinnamoylglycine and Dihydromyricetin, while *g_Ruminoclostridium 5* showed extensive negative correlations with the majority of metabolites.

Specifically, the *g_Klebsiella* showed a significant positive correlation with 3-hydroxycapric acid and 3-hydroxy-7-methoxyflavone across all four groups (*p* < 0.05). The relative abundance of *g_Klebsiella* accounted for 0.004% (CL1 group), 0.062% (CL2 group), 0.038% (CL3 group), and 0.060% (CL4 group) among treatment groups, whereas it was only 0.006% for the control groups. Although dietary supplementation with the *Salvia sclarea* L. extract markedly elevated the relative abundance of *g_Klebsiella* in the higher-dose groups (*p* < 0.05), its overall proportion remained below 0.1% of the total microbiota in all experimental groups, indicating that *g_Klebsiella* did not develop into a dominant taxon.

## 4. Discussion

The gut serves not only as the central organ for digestion and absorption in lambs, but also as the body’s largest immune modulator. The complex microbial community colonizing the gut—including bacteria, fungi, and viruses—is recognized as a “microbial organ” that maintains a tight symbiotic relationship with the host [7]. Given that ruminants lack endogenous enzymes to degrade complex plant polysaccharides, the gastrointestinal microbiota undertakes critical metabolic functions. These microbes ferment plant polysaccharides (e.g., cellulose) to produce volatile fatty acids, thereby supplying energy to the host [8]. Simultaneously, they decompose plant secondary metabolites, such as tannins, flavonoids, and alkaloids, which are essential for maintaining host health. By producing beneficial metabolites and stimulating immunoglobulin secretion, the microbiota establishes a robust intestinal epithelial barrier that effectively defends against pathogen invasion [9]. However, the lamb gut is highly susceptible to external disturbances. Stressors such as weaning readily disrupt microbial homeostasis, leading to compromised barrier function, inflammatory responses, and subsequent reductions in production performance. Consequently, maintaining the stability of the gut microbial community is paramount for mitigating weaning stress, promoting healthy growth, and enhancing immunity in lambs. *Salvia sclarea* L. extract, rich in bioactive plant compounds, may regulate the structure of the microbiota and influence the metabolic transformation of polysaccharides and bioactive compounds, and ultimately regulating immune responses, nutrient absorption, and growth development in lambs [5]. This offers a theoretical basis for developing green, efficient feed additives to alleviate weaning stress and promote healthy lamb production.

To investigate the effects of *Salvia sclarea* L. extract supplementation on the microbiota of lambs, 16S rDNA sequencing was performed on fecal samples. Analysis of community richnes (Chao1 and ACE indices) and diversity (Shannon and Simpson indices) revealed no significant differences between the *Salvia sclarea* L. extract treatment groups (CL1–CL4 group) and the CK group, indicating that *Salvia sclarea* L. extract had no significant impact on the Alpha-diversity of the microbiota in lambs. Further analysis of the top ten phyla and genera by relative abundance demonstrated high compositional similarity across treatment groups. Firmicutes, Bacteroidetes, and Spirochaetes were identified as the dominant phyla in all groups, constituting the core microbiota of the lamb gut in this study. Extensive research has documented the ubiquitous distribution of Firmicutes and Bacteroidetes across mammalian gastrointestinal tracts. These phyla constitute the core components of the gastrointestinal microbiota in ruminants, playing crucial ecological and functional roles [10]. Specifically, Firmicutes primarily participate in fiber degradation and short-chain fatty acid production [11], whereas Bacteroidetes play pivotal roles in the catabolism of carbohydrates, lipids, and proteins [12]. In addition, Spirochaetes are closely associated with lipid metabolic pathways [13] and possess the capacity to degrade pectin and xylan [14]. In the present study, Spirochaetes were identified as one of the dominant phyla. Following *Salvia sclarea* L. extract supplementation, its relative abundance exhibited an increasing trend toward increase to varying degrees, suggesting that the extract may influence host lipid metabolism by modulating the abundance of this phylum. Meanwhile, we observed that the abundance of Verrucomicrobia in the CL3 and CL4 groups was significantly higher than that in the CL1 and CL2 groups. As a low-abundance yet functionally significant member of the gut microbiota, Verrucomicrobia is represented by the genus Akkermansia, which is renowned for mucin degradation and exerts beneficial effects through reinforcing the gut mucus barrier and modulating host immune responses [15]. The abundance of Verrucomicrobia is positively correlated with host metabolic health, and its increase may indicate enhanced gut barrier function [16]. This finding suggests that medium-to-high concentrations of *Salvia sclarea* L. extract may improve mucosal barrier integrity in lambs by selectively promoting the proliferation of Verrucomicrobia, thereby alleviating weaning stress-induced gut dysfunction. Tenericutes, as colonizing bacteria of the gut mucosa, primarily participate in mucosal homeostasis regulation [17]. In this study, the relative abundance of Tenericutes in all treatment groups showed a decreasing trend compared to the CK group, indicating that *Salvia sclarea* L. extract has a certain inhibitory effect on this type of mucosa-associated bacteria. Notably, members of Tenericutes lack cell walls, enabling them to tightly adhere to host cell surfaces and evade immune recognition; their overgrowth or proliferation under compromised host immunity can trigger chronic inflammation, gut barrier damage, and associated pathological changes [18]. Therefore, the reduced abundance of this phylum may reflect the inhibitory effect of *Salvia sclarea* L. extract on potential opportunistic pathogens, contributing to decreased mucosal inflammation risk and maintenance of gut barrier integrity.

Among the top ten genera by relative abundance, *Ruminococcaceae* occupied a substantial proportion, including genera such as *Ruminococcaceae UCG-005, UCG-010, UCG-014*, and *NK4A214 group*. As key cellulolytic bacteria in the ruminant gut, members of this genus participate in the catabolism of plant polysaccharides by secreting cell wall-degrading enzymes and producing butyrate, which provides energy for the host and maintains colonic epithelial health [19]. In this study, *Ruminococcaceae UCG-010* exhibited a relatively higher abundance in the CL4 group, while *Ruminococcaceae UCG-014* showed the highest abundance in the CL1 group. These variation trends reflect the impact of different concentrations on the stability of gut fiber fermentation function. Additionally, the *Rikenellaceae RC9 gut group*, which possesses polysaccharide-degrading capabilities, can ferment unabsorbed carbohydrates to produce short-chain fatty acids including acetate, propionate, and butyrate [20]. Similar to the dose-specific modulation reported for Zanthoxylum bungeanum essential oil (EOZB) by Zhang et al. [21], wherein 10 mL/kg EOZB significantly elevated *Ruminococcaceae* and *Rikenellaceae* abundances in lamb, our study observed comparable enrichments of these taxa under *Salvia sclarea* L. extract supplementation. This convergence across distinct phytogenic sources (Zanthoxylum vs. *Salvia sclarea* L. extract) suggests that plant essential oils may share a conserved mechanism in promoting cellulolytic and polysaccharide-degrading communities, likely mediated by their common terpenoid and phenolic constituents. In the present study, the impact of *Salvia sclarea* L. extract on the *Rikenellaceae RC9 gut group* exhibited a distinct concentration-dependent pattern. While abundance increased to varying degrees in the CL1–CL3 groups, it declined in the CL4 group. This suggests that optimal concentrations of *Salvia sclarea* L. extract may promote the proliferation and metabolic function of this core functional group—enhancing carbohydrate fermentation and short-chain fatty acid production—whereas excessive active components at higher concentrations may exert inhibitory effects. The *Christensenellaceae R-7 group*, belonging to the phylum Firmicutes, represents a highly heritable, mucosa-associated bacterial lineage. Research has demonstrated that this group contributes to gut barrier integrity through the formation of protective biofilms; consequently, its enrichment is regarded as a hallmark of gut homeostasis and robust host immune tolerance [22]. Furthermore, *Christensenellaceae R-7 group* plays a pivotal role in the degradation of fibrous materials [23]. In this study, the relative abundance of the *Christensenellaceae R-7 group* showed some fluctuation among the treatment groups. Compared with the CK group, the abundance in the CL3 group showed an increasing trend, implying that appropriate supplementation of *Salvia sclarea* L. extract may enhance gut barrier function and fiber metabolic capacity.

The gut microbiota provides a crucial defensive barrier against the invasion of pathogenic microorganisms [24]. Serum immunoglobulins (IgA, IgM, and IgG) and cytokines (IL-1β, IL-6, and TNF-α) have been extensively employed as indicators for evaluating host immune function [25]. Previous studies have demonstrated that gut microbes can indirectly influence host immune homeostasis by regulating immunoglobulin production and modulating the levels of inflammatory cytokines in the gut or circulation [26]. Plant extracts possess anti-inflammatory and immunomodulatory activities [27], and multiple bioactive components in *Salvia sclarea* L. extract have been confirmed to exhibit pharmacological effects including antimicrobial, anti-inflammatory, and antioxidant properties. Following supplementation with *Salvia sclarea* L. extract the levels of serum immunoglobulins (IgA, IgG, and IgM) and anti-inflammatory cytokines were significantly elevated, suggesting that it enhances the systemic immune capacity of lambs [5]. Through correlation analysis, we identified microorganisms significantly associated with host immune indices. The results revealed that core bacteria, including the *Christensenellaceae R-7 group* and *Ruminococcaceae UCG-005,* exhibited negative correlations with specific immune parameters, implying that *Salvia sclarea* L. extract may modulate systemic immune responses in lambs by reshaping gut microbiota structure.

Pyrimidine metabolism, a vital biochemical process in living organisms, involves the synthesis and degradation of pyrimidine nucleotides [28]. As key structural components for diverse cellular functions, pyrimidine nucleotides play a crucial role in the synthesis of DNA, RNA, glycoproteins, and phospholipids [29]. This study found that dietary supplementation with *Salvia sclarea* L. extract significantly reshaped nucleotide metabolism in lamb serum. Specifically, serum pyrimidine metabolites (such as N4-Acetylcytidine, 2′-deoxycytidine, and Thymidine) were significantly reduced, reflecting the regulation of the pyrimidine nucleotide synthesis pathway by the extract. We speculate that *Salvia sclarea* L. extract. may trigger cellular metabolic reprogramming, reallocating resources away from non-essential nucleotide accumulation to optimize energy utilization efficiency. In sharp contrast, purine metabolism-related metabolites, such as Allopurinol riboside and Inosine, showed a significant increase. Among these, Allopurinol riboside, a metabolite of allopurinol, can competitively inhibit the degradation of Inosine by purine nucleoside phosphorylase, leading to an increase in Inosine content [30]. Inosine, which was significantly upregulated in the CL2 group, serves as a key metabolite in purine biosynthesis and degradation. It not only participates in the body’s material and energy metabolism [31], but has also been confirmed to be an immunomodulatory molecule with broad biological activity. Early studies have demonstrated that Inosine exhibits neuroprotective and immunomodulatory effects in various experimental models, with its beneficial effects typically mediated by regulating oxidative stress and inflammatory responses [32]. Dietary interventions that modulate gut microbiota (e.g., enriching Lactobacillus) promote Inosine accumulation in colonic epithelial cells, thereby exerting anti-inflammatory effects; meanwhile, direct Inosine supplementation enhances serum antioxidant capacity and protects against gut injury in mice [32]. Exogenous Inosine conferred similar protective effects in colitis models by activating A_2A_ receptors and Peroxisome Proliferator-Activated Receptor (PPAR)-γ signaling pathways [33]. These findings suggest that as a bioactive molecule, Inosine plays a unique protective role in mitigating inflammation associated with immunodeficiency and autoimmune diseases by regulating immune homeostasis. In contrast, the increased levels of pyrimidine metabolites (e.g., 2′-deoxycytidine and Deoxyuridine) observed in the CK group may indicate pyrimidine metabolic dysregulation, potentially compromising cellular energy metabolism and signal transduction. Dietary proteins are degraded by gastrointestinal microbiota into nitrogenous small molecules, including purine and pyrimidine nucleotides [34]. These nucleotides can be derived from purine nucleosides (e.g., Inosine and Guanosine) through de novo synthesis and salvage pathways, and subsequently degraded into purine bases (e.g., xanthine, hypoxanthine, guanine, and adenine), participating in energy metabolism and metabolic regulation [35]. In this experiment, serum nucleoside metabolites (Guanosine, Adenosine, and Inosine) were significantly elevated in the *Salvia sclarea* L. groups, indicating enhanced purine metabolic pathway activity, which may accelerate protein catabolism and promote metabolism. These findings demonstrate that *Salvia sclarea* L. extract not only influences gut microbiota structure but may also affect immune responses and energy metabolism by regulating host nucleotide metabolic pathways. Serum lipid metabolites associated with the Retrograde endocannabinoid signaling pathway were significantly altered in lambs supplemented with *Salvia sclarea* L. extract. Specifically, levels of fatty amides (Oleamide, Erucamide) and certain medium-chain fatty acids (Capric acid) were elevated, while some saturated fatty acids (Octadecanoic acid, Chaulmoogric acid) were reduced. It is also enriched with various unsaturated fatty acids, including linoleic acid and α-linolenic acid. These exogenous fatty acids can be directly absorbed and utilized by the animal or converted into polyunsaturated fatty acids (PUFAs) through the biosynthesis of unsaturated fatty acids pathway, exerting multiple beneficial effects in the animal body [36]. Studies have shown that PUFAs modulate various functions of both innate and adaptive immunity. They can be incorporated into cell membrane phospholipids, altering membrane fluidity, lipid raft structure, and signal molecule localization [37]. They also serve as precursors for eicosanoids to regulate inflammatory responses and activate nuclear receptors (PPARs) to regulate gene expression, thereby influencing cell signaling [38]. Beyond immunomodulation, PUFAs play critical roles in energy metabolism. As precursors for bioactive molecule synthesis, they constitute stable fatty acyl moieties within triglycerides for efficient energy storage [39]. During periods of increased energy demand, fatty acyl groups are hydrolyzed to release fatty acids and glycerol; the former undergo β-oxidation to generate ATP, while the latter is converted to glucose to participate in glucose metabolism [40]. Concurrently, metabolites in the retrograde endocannabinoid signaling pathway, such as Anandamide (AEA) and 2-Arachidonoylglycerol (2-AG), are known to modulate neurotransmitter release by activating cannabinoid receptors (CB_1_ or CB_2_) [41]. In this study, L-Glutamate levels were significantly elevated in the CL2, CL3, and CL4 groups, accompanied by enhanced serum immune and antioxidant capacities. Previous studies have identified L-Glutamate as a critical node linking amino acid metabolism with energy metabolism. It can be converted into α-Ketoglutarate through the glutamate-pyruvate transamination reaction, subsequently entering the tricarboxylic acid (TCA) cycle to generate NADH and FADH_2_ for ATP synthesis, thereby providing energy support for the body [42]. Furthermore, Glutamate serves as the rate-limiting substrate for the synthesis of Glutathione (GSH). As the predominant endogenous antioxidant in intestinal epithelial cells, GSH effectively scavenges reactive oxygen species, thereby preserving gut barrier integrity. Consequently, the elevated L-Glutamate levels observed not only reflect active energy metabolism but also suggest a pivotal role in gastrointestinal antioxidant defense by enhancing GSH synthesis.

## 5. Conclusions

This study demonstrates that dietary supplementation with *Salvia sclarea* L. extract alters the composition of the microbiota in lambs. At the genus level, the relative abundance of major genera was closely associated with host immune indices. Meanwhile, *Salvia sclarea* L. extract significantly modulated Purine metabolism, Pyrimidine metabolism and oxidative stress-related signaling pathways in lambs. Integrated analysis of microbiome and metabolome further revealed significant interrelationships between alterations in microbial community structure and host metabolic responses, providing a theoretical basis for the application of *Salvia sclarea* L. extract as a functional feed additive in ruminant production. Comprehensive results indicated that the supplemental dosage of 0.12 mL/kg *Salvia sclarea* L. extract (CL3 group) showed the best regulatory effect, which could be regarded as the optimal addition level.

## Figures and Tables

**Table 1:** Composition and nutrient levels of the basal diet (DM basis, %).

Ingredient	Content	Nutrition Level ^(^^2)^	Content
Corn	27.2	ME/(MJ/Kg)	12.74
Soybean meal	14.9	CP	15.43
NaCl	0.5	DM	42.11
Alfalfa Hay	21.5	NDF	33.75
Silage Corn	35.5	ADF	30.23
Premix ^(1)^	0.4	Ca	0.28
Total	100	P	0.23

Note: ^(1)^ Premix is provided per kilogram of feed. Each kilogram of trace element premix contains 5 mg of CuSO_4_·5H_2_O, 30 mg of FeSO_4_·7H_2_O, 20 mg of MnSO_4_·5H_2_O, 20 mg of ZnSO_4_·7H_2_O, 20 mg of KI, 40 mg of Na_2_SeO_3_, and 50 mg of CoCl·6H_2_O. VA 3000 IU, VD 400 IU, VE 90 IU. ^(2)^ Metabolic energy is calculated according to NRC [6], while other indicators are measured values.

## Data Availability

The data presented in this study are available on request from the corresponding author. The datasets presented in this article are not readily available because the data are part of an ongoing study or due to time limitations.

## Supplementary Materials

The following supporting information can be downloaded at: https://www.mdpi.com/article/10.3390/microorganisms14051163/s1. Table S1: Statistical Results of Raw Data Quality Control.

## References

[1] Wang Z., Zhao Y., Fan D., Zhang J., Diao Q., Cui K. (2025). Sheep-Derived *Lactobacillus johnsonii* M5 Enhances Immunity and Antioxidant Capacity, Alleviates Diarrhea, and Improves Intestinal Health in Early-Weaned Lambs. Microorganisms.

[2] Huo G., Liu X., Huo J., Feng J., Zhao X., Li Y., Wang B., Zhao J. (2026). Maternal Dietary Taurine Supplementation Improves Intestinal Health of Lambs via Modulating Gut Microbiota and Barrier Function. Front. Microbiol..

[3] Sanz Y., Cryan J.F., Deschasaux-Tanguy M., Elinav E., Lambrecht R., Veiga P. (2025). The Gut Microbiome Connects Nutrition and Human Health. Nat. Rev. Gastroenterol. Hepatol..

[4] Zhussupova A., Zhumaliyeva G., Ogay V., Issabekova A., Ross S.A., Zhusupova G.E. (2022). Immunomodulatory Effects of Plant Extracts from *Salvia deserta* Schang. and *Salvia sclarea* L.. Plants.

[5] Ma X., Niu Y., Nan S., Zhang W. (2024). Effect of *Salvia sclarea* L. Extract on Growth Performance, Antioxidant Capacity, and Immune Function in Lambs. Front. Vet. Sci..

[6] National Research Council (1975). Nutrient Requirements of Sheep.

[7] Allin K.H., Nielsen T., Pedersen O. (2015). Mechanisms in Endocrinology: Gut Microbiota in Patients with Type 2 Diabetes Mellitus. Eur. J. Endocrinol..

[8] Henderson G., Cox F., Ganesh S., Jonker A., Young W., Abecia L., Angarita E., Aravena P., Nora Arenas G., Global Rumen Census Collaborators (2015). Rumen Microbial Community Composition Varies with Diet and Host, but a Core Microbiome Is Found across a Wide Geographical Range. Sci. Rep..

[9] Šigutová H., Pyszko P., Šigut M., Czajová K., Kostovčík M., Kolařík M., Hařovská D., Drozd P. (2024). Concentration-Dependent Effect of Plant Secondary Metabolites on Bacterial and Fungal Microbiomes in Caterpillar Guts. Microbiol. Spectr..

[10] Li B., Jia G., Wen D., Zhao X., Zhang J., Xu Q., Zhao X., Jiang N., Liu Z., Wang Y. (2022). Rumen Microbiota of Indigenous and Introduced Ruminants and Their Adaptation to the Qinghai–Tibetan Plateau. Front. Microbiol..

[11] Kong L., Wang B., Wang Y., Hu R., Atiewin A., Gao D., Gao Y., Ma H. (2019). Characterization of Bacterial Community Changes and Antibiotic Resistance Genes in Lamb Manure of Different Incidence. Sci. Rep..

[12] Jami E., Israel A., Kotser A., Mizrahi I. (2013). Exploring the Bovine Rumen Bacterial Community from Birth to Adulthood. ISME J..

[13] Yang T., Jiang Y., Tang J., Chang G., Zhao W., Hou S., Chen G. (2022). Comparison of Cecal Microbiota and Performance Indices Between Lean-Type and Fatty-Type Pekin Ducks. Front. Microbiol..

[14] Tokuda G., Mikaelyan A., Fukui C., Matsuura Y., Watanabe H., Fujishima M., Brune A. (2018). Fiber-Associated Spirochetes Are Major Agents of Hemicellulose Degradation in the Hindgut of Wood-Feeding Higher Termites. Proc. Natl. Acad. Sci. USA.

[15] Li Y., Huang Y., Liang H., Wang W., Li B., Liu T., Huang Y., Zhang Z., Qin Y., Zhou X. (2023). The Roles and Applications of Short-Chain Fatty Acids Derived from Microbial Fermentation of Dietary Fibers in Human Cancer. Front. Nutr..

[16] Cani P.D., Depommier C., Derrien M., Everard A., De Vos W.M. (2022). Akkermansia Muciniphila: Paradigm for next-Generation Beneficial Microorganisms. Nat. Rev. Gastroenterol. Hepatol..

[17] Luchan J., Choi C., Carrier R.L. (2021). Reactive Oxygen Species Limit Intestinal Mucosa-Bacteria Homeostasis In Vitro. Sci. Rep..

[18] Wang Y., Huang J.-M., Zhou Y.-L., Almeida A., Finn R.D., Danchin A., He L.-S. (2020). Phylogenomics of Expanding Uncultured Environmental Tenericutes Provides Insights into Their Pathogenicity and Evolutionary Relationship with Bacilli. BMC Genom..

[19] Anderson C.J., Koester L.R., Schmitz-Esser S. (2021). Rumen Epithelial Communities Share a Core Bacterial Microbiota: A Meta-Analysis of 16S rRNA Gene Illumina MiSeq Sequencing Datasets. Front. Microbiol..

[20] Pang K., Wang J., Chai S., Yang Y., Wang X., Liu S., Ding C., Wang S. (2024). Ruminal Microbiota and Muscle Metabolome Characteristics of Tibetan Plateau Yaks Fed Different Dietary Protein Levels. Front. Microbiol..

[21] Zhang H., Lang X., Li X., Chen G., Wang C. (2022). Effect of *Zanthoxylum bungeanum* essential oil on rumen enzyme activity, microbiome, and metabolites in lambs. PLoS ONE.

[22] Waters J.L., Ley R.E. (2019). The Human Gut Bacteria Christensenellaceae Are Widespread, Heritable, and Associated with Health. BMC Biol..

[23] Evans N.J., Brown J.M., Murray R.D., Getty B., Birtles R.J., Hart C.A., Carter S.D. (2011). Characterization of Novel Bovine Gastrointestinal Tract *Treponema* Isolates and Comparison with Bovine Digital Dermatitis Treponemes. Appl. Environ. Microbiol..

[24] Khan I., Bai Y., Zha L., Ullah N., Ullah H., Shah S.R.H., Sun H., Zhang C. (2021). Mechanism of the Gut Microbiota Colonization Resistance and Enteric Pathogen Infection. Front. Cell. Infect. Microbiol..

[25] Yin X., Ji S., Duan C., Tian P., Ju S., Yan H., Zhang Y., Liu Y. (2022). Dynamic Change of Fungal Community in the Gastrointestinal Tract of Growing Lambs. J. Integr. Agric..

[26] Geng S.-T., Zhang Z.-Y., Wang Y.-X., Lu D., Yu J., Zhang J.-B., Kuang Y.-Q., Wang K.-H. (2020). Regulation of Gut Microbiota on Immune Reconstitution in Patients with Acquired Immunodeficiency Syndrome. Front. Microbiol..

[27] An Q., Ren J.-N., Li X., Fan G., Qu S.-S., Song Y., Li Y., Pan S.-Y. (2021). Recent Updates on Bioactive Properties of Linalool. Food Funct..

[28] Kim Y.J., Shin Y.K., Seo E., Seol G.H. (2022). Astrocytes Reduce Store-Operated Ca^2+^ Entry in Microglia under the Conditions of an Inflammatory Stimulus and Muscarinic Receptor Blockade. Pharmaceuticals.

[29] Bellin L., Melzer M., Hilo A., Garza Amaya D.L., Keller I., Meurer J., Möhlmann T. (2023). Nucleotide Limitation Results in Impaired Photosynthesis, Reduced Growth and Seed Yield Together with Massively Altered Gene Expression. Plant Cell Physiol..

[30] Worledge C.S., Kostelecky R.E., Zhou L., Bhagavatula G., Colgan S.P., Lee J.S. (2024). Allopurinol Disrupts Purine Metabolism to Increase Damage in Experimental Colitis. Cells.

[31] Kannangara D.R.W., Roberts D.M., Furlong T.J., Graham G.G., Williams K.M., Day R.O. (2012). Oxypurinol, Allopurinol and Allopurinol-1-riboside in Plasma Following an Acute Overdose of Allopurinol in a Patient with Advanced Chronic Kidney Disease. Br. J. Clin. Pharmacol..

[32] Yasutake Y., Tomita K., Higashiyama M., Furuhashi H., Shirakabe K., Takajo T., Maruta K., Sato H., Narimatsu K., Yoshikawa K. (2017). Uric Acid Ameliorates Indomethacin-induced Enteropathy in Mice through Its Antioxidant Activity. J. Gastroenterol. Hepatol..

[33] Guinzberg R., Cortés D., Díaz-Cruz A., Riveros-Rosas H., Villalobos-Molina R., Piña E. (2006). Inosine Released after Hypoxia Activates Hepatic Glucose Liberation through A_3_ Adenosine Receptors. Am. J. Physiol. Endocrinol. Metab..

[34] Kasahara K., Kerby R.L., Zhang Q., Pradhan M., Mehrabian M., Lusis A.J., Bergström G., Bäckhed F., Rey F.E. (2023). Gut Bacterial Metabolism Contributes to Host Global Purine Homeostasis. Cell Host Microbe.

[35] Tran D.H., Kim D., Kesavan R., Brown H., Dey T., Soflaee M.H., Vu H.S., Tasdogan A., Guo J., Bezwada D. (2024). De Novo and Salvage Purine Synthesis Pathways Across Tissues and Tumors. Cell.

[36] Xu P., Wang K., Lu C., Dong L., Gao L., Yan M., Aibai S., Yang Y., Liu X. (2017). The Protective Effect of Lavender Essential Oil and Its Main Component Linalool against the Cognitive Deficits Induced by D-Galactose and Aluminum Trichloride in Mice. Evid.-Based Complement. Altern. Med..

[37] Shaikh S.R., Jolly C.A., Chapkin R.S. (2012). N-3 Polyunsaturated Fatty Acids Exert Immunomodulatory Effects on Lymphocytes by Targeting Plasma Membrane Molecular Organization. Mol. Asp. Med..

[38] Lu H.-C., Mackie K. (2016). An Introduction to the Endogenous Cannabinoid System. Biol. Psychiatry.

[39] Park J., Choi J., Kim D.-D., Lee S., Lee B., Lee Y., Kim S., Kwon S., Noh M., Lee M.-O. (2021). Bioactive Lipids and Their Derivatives in Biomedical Applications. Biomol. Ther..

[40] Chandel N.S. (2021). Lipid Metabolism. Cold Spring Harb. Perspect. Biol..

[41] Marques B.L., Lirio P.H.C., Vicente M.A., Unzueta-Larrinaga P., Urigüen L., Campos A.C. (2025). Cannabinoids and Extracellular Vesicles as Potential Biomarkers and Therapeutic Targets in Neuropsychiatric Disorders: A Hypothesis-Driven Review. Pharmaceuticals.

[42] De Pablo M.A., De Cienfuegos G.Á. (2000). Modulatory Effects of Dietary Lipids on Immune System Functions. Immunol. Cell Biol..

