# Supplementary material for: Effects of Dietary Salvia sclarea L. Extract Supplementation on the Gut Microbiota, and Serum Metabolome in Lambs
Source: Microorganisms. 2026 May 21;14(5):1163. doi: 10.3390/microorganisms14051163 (PMC13210186; doi:10.3390/microorganisms14051163)
Supplement: Supplementary file 1 [file microorganisms-14-01163-s001.zip › microorganisms-4301252-supplementary.pdf]

Supplementary Table S1. Statistical Results of Raw Data Quality Control

SAMPLE	READS	BASES	Q20	Q20RATE	Q30	Q30RATE	GC
CK_1	92180	37942058	37315877	98.35	35909602	94.64	20132126
CK_2	106737	43747701	43085824	98.49	41561817	95.00	23316540
CK_3	93774	38659335	38023638	98.36	36602847	94.68	20428660
CK_4	96523	39638626	38998593	98.39	37564862	94.77	21016703
CK_5	105850	43571691	42893061	98.44	41339819	94.88	23094454
CK_6	99804	40916937	40271377	98.42	38823045	94.88	21855358
CK_7	108161	44475647	43780407	98.44	42195632	94.87	23635427
CK_8	104990	43094058	42409410	98.41	40866104	94.83	22992041
CL1_1	111123	45707225	44974738	98.40	43316287	94.77	24224920
CL1_2	116686	47765156	47022024	98.44	45340914	94.92	25532485
CL1_3	111814	46145208	45391924	98.37	43704180	94.71	24399996
CL1_4	114931	47090930	46314205	98.35	44585589	94.68	25117791
CL1_5	111312	45647149	44910005	98.39	43262526	94.78	24285605
CL1_6	116780	47962234	47189623	98.39	45471332	94.81	25404534
CL1_7	84204	34666198	34128714	98.45	32900216	94.91	18467854
CL1_8	105696	43363239	42659954	98.38	41082040	94.74	22973979
CL2_1	97003	40028064	39362431	98.34	37879167	94.63	21135405
CL2_2	90450	37147155	36558022	98.41	35242448	94.87	19739761
CL2_3	92745	38135465	37518943	98.38	36143509	94.78	20311588
CL2_4	93354	38404011	37754438	98.31	36312742	94.55	20338220
CL2_5	97805	40204879	39558764	98.39	38111651	94.79	21392316
CL2_6	88077	36317900	35725323	98.37	34408706	94.74	19222893
CL2_7	88258	36306529	35727257	98.40	34421958	94.81	19229860
CL2_8	89449	36763229	36143117	98.31	34769355	94.58	19647782
CL3_1	96654	39818141	39209936	98.47	37825919	95.00	21048157
CL3_2	107312	44038398	43323935	98.38	41731905	94.76	23435927
CL3_3	102354	42219631	41520858	98.34	39965745	94.66	22459407
CL3_4	10140	41762369	41051492	98.30	39468701	94.51	22247433

	6						
CL3_5	98573	40553130	39872434	98.32	38355115	94.58	21534192
CL3_6	108818	44778260	44041499	98.35	42398295	94.68	23759756
CL3_7	112154	45930555	45190977	98.39	43549242	94.82	24545912
CL3_8	101519	41604426	40907037	98.32	39369770	94.63	22237349
CL4_1	105574	43497778	42819955	98.44	41283805	94.91	23010342
CL4_2	105155	43185089	42503034	98.42	40965596	94.86	22895660
CL4_3	111464	45782156	45050806	98.40	43407771	94.81	24379807
CL4_4	121751	49969785	49147729	98.35	47313304	94.68	26448451
CL4_5	80113	33007912	32487974	98.42	31299806	94.83	17462293
CL4_6	106811	44055427	43320183	98.33	41676786	94.60	23068607
CL4_7	95869	39271837	38642793	98.40	37232769	94.81	20890862
CL4_8	103480	42368142	41653251	98.31	40085624	94.61	22640546
